# Rationally engineered polymeric interfaces for selective and dynamic recognition of vesicular agents

**DOI:** 10.1002/2211-5463.70365

**Published:** 2026-11-12

**Authors:** Özgecan Erdem, Eylul Gulsen Yilmaz, Selcan Genc, Nil Kılıç, Fatih Inci

**Affiliations:** ^1^ UNAM—National Nanotechnology Research Center Bilkent University Ankara Turkey; ^2^ Institute of Materials Science and Nanotechnology Bilkent University Ankara Turkey

**Keywords:** biosensors, extracellular vesicles, Molecularly imprinted polymers, vesicular agents, viruses

## Abstract

It is exceedingly hard to accurately identify and separate nanoscale vesicular entities such as extracellular vesicles (EVs), lipid‐enveloped and membrane‐associated viral particles, and lipid‐based drug delivery nanoparticles. These systems have a membrane‐bound structure, but their surface composition, mechanical properties, and molecular structures are diverse. This makes it challenging to differentiate them in complicated biological matrices. Molecularly imprinted polymers (MIPs) are synthetic recognition materials that solve this problem by being selective, stable, and cost effective. This review provides a thorough analysis of rationally engineered MIP interfaces for the targeted detection of vesicular agents. We first analyze the physicochemical properties of vesicular surfaces; including lipid bilayer composition, surface protein and glycan profiles, charge distribution, and membrane mechanical characteristics. We then examine how these attributes guide the strategic design of imprinted recognition interfaces. We critically examine the recognition strategies and supporting material architectures relevant to the molecular imprinting of soft and heterogeneous vesicular templates, including surface and epitope imprinting, as well as core–shell nanoparticle and cryogel‐based macroporous architectures. This is done with a specific focus on the design of recognition‐site accessibility, membrane‐associated template selection, and material architectures adapted to soft vesicular targets at the recognition interface. The integration of vesicle MIPs into analytical and sensing systems is examined across optical, electrochemical, membrane‐based, and microfluidic formats, including applications in EV subpopulation profiling, virus detection, and targeting concepts relevant to lipid‐based vesicular carriers. Finally, we evaluate the current limitations and potential avenues for next‐generation MIPs that provide improved selectivity, sensitivity, and clinical applicability for vesicular biomarker diagnostics.

## Graphical abstract

This review explores how molecularly imprinted polymers can be engineered to recognize complex nanoscale vesicles, including extracellular vesicles and membrane‐enveloped viruses. By tailoring polymer cavities to vesicle surface chemistry and structure, these synthetic receptors support selective detection, separation, and integration with optical, electrochemical, membrane‐based, and microfluidic platforms for future bioanalytical applications.

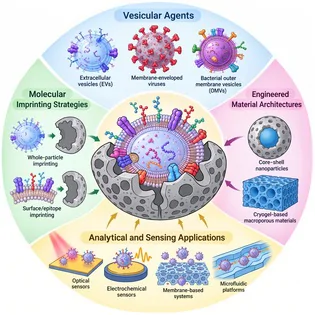

**Abbreviations**
ACE2: angiotensin‐converting enzyme 2
AFM: atomic force microscopy
APBA: 3‐aminophenylboronic acid
EIS: electrochemical impedance spectroscopy
EV: extracellular vesicle
HAV: hepatitis A virus
HER2: Human Epidermal Growth Factor Receptor 2
HEV: hepatitis E virus
HIV‐1: Human Immunodeficiency Virus Type 1
LNP: lipid nanoparticle
ILV: intraluminal vesicle
LOD: limit of detection
LPS: lipopolysaccharide
MIP: molecularly imprinted polymer
MISEV2023: Minimal Information for Studies of Extracellular Vesicles 2023
MRPS: microfluidic resistive pulse sensing
MV: microvesicle
MVB: multivesicular body
NIP: non‐imprinted polymer
NSCLC: non‐small cell lung cancer
OMV: outer membrane vesicle
PEG: polyethylene glycol
PFU: Plaque‐forming unit
POCT: point‐of‐care testing
QSPR: Quantitative Structure–Property Relationship
SARS‐CoV‐2: Severe Acute Respiratory Syndrome Coronavirus 2
SELEX: Systematic Evolution of Ligands by EXponential Enrichment
SPR: surface plasmon resonance

Selective recognition and differentiation of nanoscale vesicular particles remain major challenges in bioanalytical science and nanomedicine. Vesicular agents mediate biological communication and support biomedical applications by transporting functional biomolecules through complex biological environments. These systems include extracellular vesicles (EVs), lipid‐enveloped viruses, membrane‐associated or quasi‐enveloped viral particles, and lipid‐based drug delivery platforms [1]. Although they differ substantially in their biological origin and function, they can share membrane‐associated physicochemical features, including nanoscale dimensions, lipid‐rich interfaces, surface proteins, glycans, and charge distributions [2, 3]. In the context of this review, the term “vesicular agents” therefore refers primarily to membrane‐bound nanoscale entities. Bare non‐enveloped viruses fall outside this definition and are discussed only selectively where capsid imprinting offers a useful comparison with membrane‐oriented recognition strategies [4]. The rapidly expanding analytical and biomedical interest in extracellular vesicles, membrane‐enveloped viruses, and synthetic lipid‐based vesicles has created a growing demand for recognition systems capable of selectively distinguishing structurally complex and highly heterogeneous membrane‐bound targets. Unlike conventional small‐molecule or protein templates, vesicular agents combine nanoscale dimensions with multicomponent, dynamic, and often compositionally similar surfaces, creating distinct challenges in template handling, recognition‐site formation, target accessibility, and rebinding [5]. These characteristics make the direct translation of conventional molecular imprinting concepts to vesicular targets non‐trivial and highlight the need for imprinting strategies specifically adapted to soft, nanoscale, membrane‐bound structures. At the same time, the increasing importance of vesicular agents in liquid biopsy, infectious disease diagnostics, drug delivery, and nanomedicine makes the development of robust synthetic recognition systems particularly timely (Fig. 1).

**Fig. 1:**
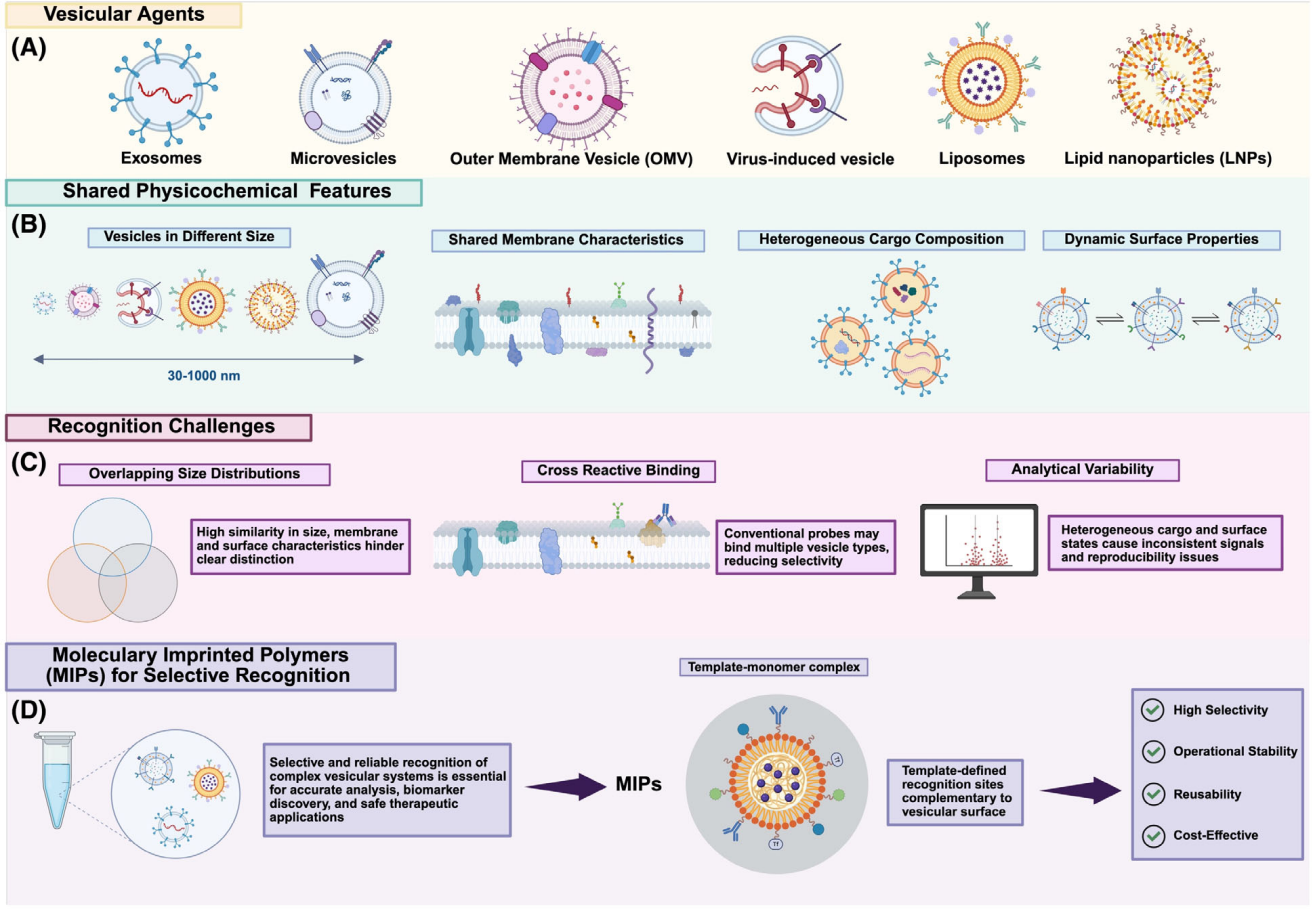
Physicochemical features and recognition challenges of vesicular agents. (A) Representative vesicular agents and (B) Shared physicochemical features. (C) Challenges limiting selective recognition and differentiation. (D) Template‐defined binding sites in MIPs for selective vesicular recognition. Created in BioRender. (https://BioRender.com/fubjh7y) is licensed under CC BY 4.0.

Among vesicular systems, EVs are among the most extensively studied vesicular systems because of their essential roles in intercellular communication and their emerging potential as non‐invasive biomarkers [6]. Their nucleic acid, protein, and lipid cargo reflect the physiological state of the cells from which they originate [7]. Exosomes and microvesicles (MVs) are two broadly described EV classes that differ in biogenesis and typical size [8]. Exosomes are generally described as vesicles measuring 30–100 nm that originate as intraluminal vesicles (ILVs) within multivesicular bodies (MVBs), and are released when MVBs fuse with the plasma membrane [9]. MVs are typically larger, approximately 200–1000 nm in diameter, and form through outward budding and fission of the plasma membrane [10]. However, overlapping size distributions and similar membrane compositions make these populations difficult to distinguish [11]. Vesicle‐mediated communication also extends beyond eukaryotic cells [12]. Outer membrane vesicles (OMVs), released by Gram‐negative bacteria, represent another class of vesicular structures involved in intercellular communication, especially concerning host‐microbe communication, including signaling, virulence, and immune modulation. For instance, Li et al. showed that OMVs derived from *Escherichia coli* Nissle 1917 can be separated into distinct size fractions exhibiting differential protein cargo profiles and immunomodulatory effects, thereby highlighting the functional heterogeneity of OMV subpopulations [13]. OMVs thus add further diversity to the vesicle landscape, compounding the challenge of selective recognition [14].

Within the viral domain, lipid‐enveloped viruses must be distinguished from quasi‐enveloped or EV‐associated viral particles, and bare non‐enveloped virions. Lipid‐enveloped viruses are especially relevant to the vesicular framework discussed here, as their host‐derived membranes confer substantial physicochemical similarity to EVs, including overlapping size ranges, bilayer organization, and membrane‐associated molecular features. Enveloped viruses such as HIV‐1 and SARS‐CoV‐2, in particular, closely resemble EVs in size, lipid bilayer architecture, and surface protein composition, making their discrimination from cell‐derived EVs exceptionally difficult [15]. Moreover, growing evidence indicates that non‐enveloped viruses, including hepatitis A virus (HAV) and hepatitis E virus (HEV), can also be released in, EV‐associated, membrane‐cloaked forms that confer EV‐like properties [16]. This vesicle‐mediated cloaking not only allows viruses to evade immune surveillance by shielding viral particles from neutralizing antibodies, but also blurs the boundaries between viral particles and endogenous vesicular populations [17]. In contrast, bare non‐enveloped virions lack a lipid bilayer and are therefore not considered vesicular agents within the scope of this review. Consequently, the considerable overlap in physicochemical and molecular properties between EVs and membrane‐bound viral particles poses a significant hurdle in their selective detection and differentiation in complex biological surroundings. Accordingly, molecular imprinting has emerged as a promising strategy for the selective recognition of membrane‐bound viral particles, supporting the development of synthetic receptors for structurally complex membrane‐bound targets [18].

Recently, there has been increasing attention toward lipid‐based nanoparticles (LNPs), such as liposomes, lipid micelles, and lipid‐based nanoemulsions which are promising platforms for drug delivery. However, several limitations still need to be overcome. Yao et al. have reported that liposomal systems, although widely used in cancer therapy, exhibit significant challenges related to their formulation‐dependent variability, as their size, stability, surface charge, and membrane properties are highly influenced by lipid composition and additives like polyethylene glycol (PEG). Furthermore, while liposomal formulations can improve circulation time and reduce certain toxicities, they may also introduce new adverse effects, highlighting trade‐offs in their therapeutic efficacy [19]. Several studies have further emphasized that LNP systems continue to face critical limitations in formulation and manufacturing, along with challenges related to stability, intracellular delivery efficiency, and endosomal escape [20]. In addition, achieving precise organ‐ and cell‐specific targeting remains difficult, while issues in scalability, reproducibility, and thermostability further restrict their widespread clinical application [21]. Collectively, these differences arising from formulation conditions point to a broader heterogeneity among lipid‐based vesicular systems, one that calls for recognition strategies capable of resolving subtle variations at the membrane surface. More recently, the membrane architecture of lipid‐based vesicles has itself been explored as a molecular recognition target. For example, phospholipid‐imprinted polymers have been successfully developed to discriminate membrane‐derived vesicles, demonstrating the potential of membrane‐oriented imprinting strategies for selective vesicular recognition [22].

These limitations have driven the need for advanced and highly selective recognition platforms capable of distinguishing complex vesicular systems. Although recent advances in biosensing have improved analytical sensitivity and detection capabilities [23], the selective recognition of heterogeneous and structurally similar vesicular targets remains challenging. To address these limitations, engineered polymer‐based recognition platforms have emerged as promising alternatives. Among these, molecularly imprinted polymers (MIPs) have attracted considerable attention due to their ability to overcome key limitations associated with both natural and synthetic alternatives [24, 25]. During the polymerization process, template molecules direct the formation of well‐defined binding cavities and functional groups within the polymer matrix, enabling MIPs to exhibit a high degree of selectivity and affinity toward their target structures [26, 27]. Even though antibodies remain the gold standard when it comes in molecular recognition, their susceptibility to denaturation, batch‐to‐batch variability, and high production costs pose significant challenges for widespread clinical implementation. Likewise, aptamers, while stable and customizable, require labor‐intensive SELEX (Systematic Evolution of Ligands by EXponential enrichment) processes and often exhibit reduced affinity in complex biological media [28]. By contrast, MIPs combine the programmable specificity of synthetic systems with antibody‐like recognition capabilities. These systems offer several key advantages, notably their exceptional physicochemical stability under extreme pH, temperature, and solvent conditions, which is critical for point‐of‐care tests (POCT) in resource‐limited settings [29]. In addition, their cost‐effective, template‐directed synthesis bypasses biological systems, enabling the rapid and adaptable generation of synthetic recognition interfaces for complex vesicular targets.

Their innate reusability therefore enables repeated binding–regeneration cycles with very little loss of recognition performance [30]. Importantly, these properties provide a useful foundation for the rational design of imprinted interfaces capable of recognizing the heterogeneous and dynamic surface features of vesicular targets. Taken together, these attributes position MIPs as a strong and versatile platform for the selective recognition of complex vesicular systems, including extracellular vesicles, viruses, and lipid‐based nanoparticles.

Despite the growing interest in molecular imprinting and vesicle‐based recognition, the rational design of imprinted polymer interfaces specifically tailored to the unique physicochemical and mechanical properties of vesicular targets remains insufficiently addressed. This topic is particularly timely as molecular imprinting is increasingly being extended from conventional molecular targets to soft, deformable, and structurally heterogeneous nanoscale systems, which introduce distinct challenges in template immobilization, cavity formation, and selective rebinding. In this review, we specifically focus on molecular imprinting strategies developed for the recognition, isolation, detection, and targeting of vesicular agents, encompassing extracellular vesicles, membrane‐enveloped viruses, and synthetic lipid‐based vesicular systems. Importantly, a distinction is made between the direct imprinting of intact vesicular structures and indirect recognition strategies based on vesicle‐associated membrane components, epitopes, or surface biomarkers. Studies involving isolated proteins or other non‐vesicular templates are discussed only when they provide a mechanistic or design principle directly transferable to vesicular‐agent imprinting, rather than as examples of vesicle imprinting themselves. Through this framework, we aim to identify how the structural complexity, membrane composition, nanoscale dimensions, and dynamic surface characteristics of vesicular agents influence template selection, imprinting strategy, recognition performance, and ultimately their analytical and biomedical applicability. We further discuss their integration into analytical and sensing platforms, while highlighting current challenges and future directions for the development of next‐generation vesicle‐imprinted recognition systems.

## MIPs for vesicular agents

MIPs are synthetic receptor systems that are designed to mimic biological recognition mechanisms. They contain binding sites with chemical and structural features complementary to the target analyte [31]. This complementarity enables selective recognition of a wide spectrum of analytes, ranging from simple small molecules to more complex biomacromolecules [32]. This selectivity stems from the microenvironments formed by the functional monomers organized around the template molecule during the polymerization process. These interactions between functional monomers and the target are mostly based on weak binding mechanisms such as hydrogen bonds, electrostatic forces, and van der Waals interactions [33, 34]. In a typical MIP synthesis, the template molecule is combined with functional monomers, a crosslinker, and an initiator to form a highly crosslinked polymer network [35, 36]. After the removal of the template, three‐dimensional cavities are generated that reflect the size and geometry of the target molecule [37]. These cavities can selectively rebind the target molecule, providing the basis for its analytical detection [38]. Because of structural stability and reusability, MIPs are often considered robust and cost‐effective alternatives to natural receptors, particularly in applications that require operation under varying environmental conditions [39, 40]. Recently, studies combining these polymers with different materials have increased. Combinations with nanomaterials or magnetic particles, in particular, have significantly improved both sensitivity and ease of use. Such systems are now used not only in sensors but also in fields such as water treatment, environmental analysis, and drug delivery [41]. Overall, when all these features are combined, MIPs provide a platform that can serve as an alternative to natural recognition elements. Their ability to combine robustness, selectivity, and ease of fabrication in the same structure makes them attractive for many applications [42, 43].

### Imprinting strategies and challenges for vesicular agents

MIPs were initially developed as synthetic receptors for relatively small and structurally rigid targets [44]. However, once the focus shifts to vesicular systems (i.e., liposomes, exosomes, enveloped viruses, or drug‐loaded nanocarriers), the situation becomes far less straightforward [22]. Vesicles are soft, highly deformable, nanoscale structures whose surfaces present a dynamic, heterogeneous landscape of lipids, glycoproteins, and membrane‐associated molecules [45, 46]. In addition, intact vesicles may deform or undergo surface rearrangement during polymerization and template removal, while their relatively large‐size limits access to recognition sites buried within highly crosslinked polymer matrices. Consequently, vesicle imprinting requires recognition sites to be generated predominantly at accessible interfaces while preserving the structural and chemical features of the vesicular surface. Translating the classical MIP paradigm to these complex templates has therefore required a substantial evolution of both conceptual frameworks and practical imprinting strategies [27].

When direct imprinting of an intact vesicle is experimentally difficult because of template instability, heterogeneity, or limited availability, a membrane‐associated component may instead be used as a surrogate recognition element. Phospholipid‐oriented imprinting has demonstrated that membrane‐associated lipid features can provide selective recognition of cell and exosome surfaces (Fig. 2A) [22, 47]. These approaches reduce the complexity of the template while retaining recognition of characteristic vesicular surface features. However, they should be distinguished from whole‐vesicle imprinting because the imprinted template represents only a selected component of the vesicular surface.

**Fig. 2:**
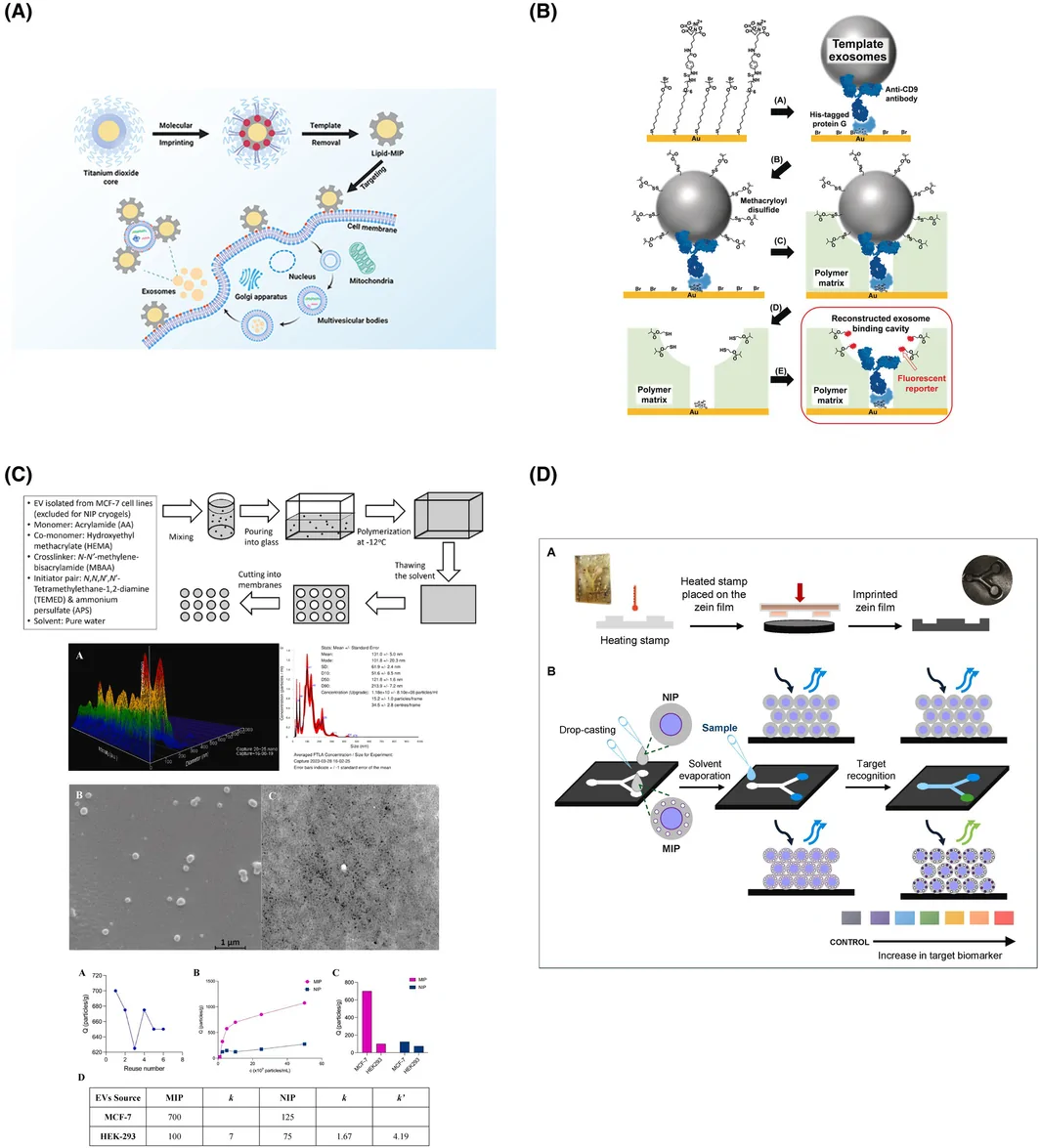
Representative molecular imprinting strategies and material architectures relevant to vesicular recognition. (A) Phospholipid imprinting for targeted cell and exosome recognition [22]. (B) Intact‐exosome molecular imprinting and post‐imprinting modification for selective exosome recognition [48]. (C) Molecularly imprinted cryogel membranes for extracellular vesicle isolation and adsorption [50]. (D) Core–shell molecularly imprinted particles on zein films for photonic detection of EV‐associated surface protein epitopes [51]. Reprinted with permission from the corresponding references.

Surface imprinting is particularly suitable for intact vesicular targets because recognition sites are formed at or close to the polymer–solution interface, minimizing diffusion into the polymer network and facilitating template removal and rebinding [48, 49]. This strategy has been successfully applied to intact extracellular vesicles, demonstrating that accessible imprinted interfaces can discriminate vesicular populations based on their surface characteristics (Fig. 2B) [48].

Macroporous cryogel architectures provide another vesicle‐compatible implementation of molecular imprinting. Their interconnected porous structure can facilitate the transport and interaction of intact vesicles while maintaining accessible recognition sites. Molecularly imprinted cryogel membranes have been demonstrated for the selective isolation and adsorption of extracellular vesicles (Fig. 2C) [50].

Core–shell molecularly imprinted particles provide a complementary architecture in which the recognition layer is confined to a thin and accessible outer region, thereby facilitating target accessibility and rebinding. This format has been applied to EV‐associated surface proteins: Vaz et al. developed silica‐core, polydopamine‐shell particles imprinted with peptide epitopes of CD81 and GLAST‐1 and integrated them into photonic zein films for label‐free detection in human serum (Fig. 2D) [51]. This study targeted peptide epitopes rather than intact vesicles, and it is therefore included as an accessible‐architecture concept transferable to vesicular targets rather than as a demonstration of intact‐EV recognition.

Overall, the central challenge in vesicle imprinting is therefore not simply the generation of geometrically complementary cavities, but the preservation and selective recognition of a soft, heterogeneous, and dynamically organized membrane interface. Vesicle‐associated membrane‐component imprinting, surface‐accessible recognition layers, macroporous cryogel architectures, and thin core–shell recognition layers represent complementary strategies for addressing these limitations. Whole‐particle viral imprinting can provide useful comparative information [52] but non‐enveloped viruses are not considered vesicular targets in this review.

### Binding kinetics of MIPs targeting vesicular agents

Insights from the imprinting of large biomacromolecular and glycoprotein targets suggest that template size, conformational flexibility, and limited accessibility can impose substantial mass‐transfer constraints [53, 54]. However, these effects have not yet been systematically quantified for intact vesicle‐imprinted systems.

These constraints are expected to be more pronounced for vesicular targets. Intact vesicles are typically one to two orders of magnitude larger than proteins and have correspondingly low diffusion coefficients (Section Rationale, mass transport considerations, and channel design), so recognition sites buried within a densely crosslinked bulk matrix are likely to remain largely inaccessible during rebinding. Consistent with this consideration, the vesicle‐imprinting studies reported to date have relied on architectures that expose recognition sites at accessible interfaces, including surface‐imprinted layers on sensor substrates [48, 49], macroporous cryogel membranes [50], and core–shell particles with thin imprinted shells [51]. In the broader MIP literature, surface imprinting [55], thin‐layer core–shell architectures [56], and interconnected porous structures [57] are established strategies for shortening diffusion paths and improving site accessibility. Whether these architectures also yield faster rebinding kinetics for intact vesicles has not yet been systematically examined, as the available vesicle‐imprinting studies primarily report sensitivity and selectivity rather than association and dissociation kinetics.

Accordingly, the kinetic performance of vesicle‐imprinted systems should be considered in terms of the combined effects of vesicle transport, recognition‐site accessibility, interfacial contact, and multivalent rebinding rather than binding affinity alone. The mechanical aspects of interfacial contact are discussed in Section Mechanical properties: Elasticity, curvature, and roughness. Systematic kinetic characterization of intact vesicle MIPs, for example through real‐time SPR measurements comparing MIP and NIP interfaces, remains an unmet open need for the field.

### Comparison with antibodies as sensing elements

MIPs aimed at vesicular agents present a compelling alternative to antibodies, which are regarded as the gold standard for molecular recognition. Antibodies developed against surface antigens of vesicular structures such as liposomes or exosomes can exhibit very high selectivity and achieve binding affinities at the subnanomolar level. Antibody production typically requires animal immunization or complex *in vitro* selection processes. Their stability is limited under extreme conditions including high temperature, organic solvents, or high pH values [58]. MIPs, on the other hand, provide a significantly different benefit due to the fact that they are fully synthetic structures [44]. It is possible to produce on a large scale, and the expenses associated with doing so are rather modest. Despite these advantages, MIPs generally exhibit greater binding‐site heterogeneity and higher levels of non‐specific adsorption than affinity‐matured antibodies, which may limit their recognition performance in complex biological matrices. Nevertheless, this difference has been significantly reduced thanks to controlled radical polymerization techniques, oriented surface imprinting approaches [59], and epitope‐based [18] strategies [60].

The emerging concept of “plastic antibodies,” in which nanoparticle MIPs functionalized with multiple epitope‐complementary cavities mimic the multivalent binding geometry of IgG antibodies, points toward a future in which the selectivity gap between biological and synthetic receptors for vesicular agents may be effectively closed [61].

## Design principles of vesicle‐imprinted MIPs

### Physicochemical features of vesicular surfaces

#### Lipid bilayer composition

The polymer must engage the vesicle's external surface, not the cargo, hence developing a polymeric recognition interface requires a detailed chemical environment analysis. The lipid bilayer underpins all surface properties in the three classes of vesicular systems discussed in this paper, but its composition and organization vary widely depending on the vesicle's biological or synthetic origin. EV membranes differ from plasma membranes. Lipidomic analyses across multiple cell lineages show that EV membranes selectively enrich cholesterol, sphingomyelin, long‐chain sphingolipids, and ether‐linked phospholipids, while exosomal membranes concentrate glycosphingolipids like hexosylceramide and lactosylceramide [62, 63]. Cholesterol organizes membrane proteins, glycolipids, and signaling molecules in lipid rafts by intercalating sphingolipid acyl chains and promotes tight lateral packing [64]. A nanoscale EV's outer surface is laterally heterogeneous due to these structured domains, providing separate areas of different composition to every approaching recognition interface. Biologically encoded lateral heterogeneity of vesicle biogenesis reflects secreting cell sorting. A well‐designed imprinted cavity should encode spatial chemical heterogeneity for a molecularly imprinted polymer to discriminate EV subpopulations. Also significant is EV lipid transbilayer organization. In the outer leaflet of the EV membrane, sphingomyelin and phosphatidylcholine have cylindrical molecular geometries that support planar bilayer packing, while phosphatidylethanolamine and phosphatidylinositol are enriched in the inner leaflet [45]. In microvesicles generated by direct plasma membrane budding, scramblase‐mediated processes move phosphatidylserine from the inner leaflet to the outside leaflet during EV formation [65]. Sialic acid‐bearing glycoproteins and glycosphingolipids on the outer bilayer surface and externalized phosphatidylserine substantially increase EVs' net negative surface charge [45]. Thus, EV surface charges depend on lipid asymmetry, glycan decorating, and protein surface structure, which are regulated by the vesicle's biogenesis history and vary predictably with cell‐of‐origin identity and physiological condition. Both EVs and enveloped viruses have a lipid bilayer with a macromolecular core, but their envelope lipids differ. The viral envelope is directed toward structured, sphingolipid‐ and cholesterol‐enriched lipid raft areas rather than the bulk plasma membrane by selectively acquiring host cell membrane lipids at the budding location [63, 66]. Compared to the bulk plasma membrane, HIV‐1 virions and exosomes from the same host cells are enriched in cholesterol, sphingomyelin, and phosphatidylserine, which explains why enveloped virions and host‐derived exosomes are physically distinct. Coronavirus spike, membrane, and envelope proteins, influenza hemagglutinin and neuraminidase, and HIV‐1 gp120/gp41 trimers are superimposed on this lipid framework [67, 68]. Enveloped viruses' outer surfaces differ from morphologically comparable EVs in their glycoproteins' spatial arrangement, glycosylation state, and surface density, which determine receptor recognition and host range. Bare non‐enveloped viruses fall outside the primary vesicular scope of this review because they lack a lipid membrane. Accordingly, capsid‐based recognition is not discussed in detail here. Where relevant, these systems are mentioned only as a conceptual contrast to membrane‐bound viral particles, whose molecular imprinting can exploit lipid‐, glycan‐, and membrane‐associated recognition features. The third class comprises lipid‐based drug delivery. An ionizable lipid becomes cationic in acidic endosomal compartments to release nucleic acids, a PEGylated lipid extends hydrophilic polyethylene glycol chains from the particle surface to enhance colloidal stability and reduce opsonization, and cholesterol maintains bilayer integrity in clinically deployed lipid nanoparticles. The PEG‐lipid molar fraction controls surface PEG chain conformation, which ranges from a collapsed mushroom‐like arrangement at low densities that partially exposes the lipid surface to an extended brush conformation at higher densities that provides steric shielding and near‐homogeneity [69]. This impacts recognition. Traditional liposomes made of zwitterionic phosphatidylcholine species have a near‐neutral surface charge under physiological conditions, but cationic lipids like DOTAP (1,2‐dioleoyl‐3‐trimethylammonium‐propane) or DODMA (1,2‐dioleyloxy‐3‐dimethylaminopropane) make them positive, and anionic lipids like DSPG (1,2‐distearoyl‐sn‐glycero‐3‐phospho‐(1′‐rac‐glycerol)) make them negative. These formulation‐dependent differences in surface charge and hydrophilicity, captured quantitatively by QSPR (Quantitative Structure–Property Relationship) modeling of liposome zeta potential [70], indicate that imprinted polymer interfaces would need to be explicitly tuned to charge dispersion and hydrophilic–lipophilic balance to distinguish between formulations—a design requirement rather than a demonstrated MIP capability at present.

#### Surface proteins, glycosylation, and charge distribution

Surface proteins and glycoconjugates provide molecular features for selective polymer recognition beyond the lipid bilayer. The EV surface proteome contains information about cellular origin and physiological state that size‐ or charge‐based capture alone cannot resolve. Tetraspanins such as CD9, CD63, and CD81 are enriched in EV populations from diverse sources and are widely used transmembrane markers in EV characterization frameworks [5, 71]. They form tetraspanin‐enriched microdomains at the vesicle surface and organize supramolecular complexes with cell‐type‐specific and pathological state‐dependent protein compositions, creating a molecular fingerprint that is replicated across cell line preparations under defined culture conditions [72]. EVs from malignant cells may display disease‐specific surface proteins such as epithelial cell adhesion molecule, epidermal growth factor receptor, HER2 (Human Epidermal growth factor Receptor 2) from HER2‐amplified breast tumors, and glypican‐1 from pancreatic cancer cells with excellent specificity [73, 74, 75, 76, 77]. Disease‐associated proteins may therefore provide candidate epitopes for designing MIPs directed toward specific EV subpopulations. However, protein‐level epitope imprinting should be distinguished from direct imprinting of intact vesicles. EVs gain chemical identity from protein glycosylation. N‐ and O‐linked glycans on exosomal surface glycoproteins form carbohydrate fingerprints that indicate secreting cell glycosylation and malignant transformation [78]. Tumor‐derived EVs derived from non‐malignant cells of the same tissue lineage due to glycosylation anomalies like increased core fucosylation, surface glycoprotein sialylation, and N‐linked glycan antennae polylactosamine branching [79]. Terminal sialic acid residues on cancer‐associated EV glycoproteins contribute a surface negative charge, steric shields over protein epitopes, and chemical handles that react reversibly with functional monomers like 3‐aminophenylboronic acid to generate boronate esters under physiological conditions [80]. This covalent imprinting chemistry records the spatial arrangement of glycan‐bearing surface proteins and the chemical nature of their carbohydrate termini, enabling recognition specificity that non‐covalent shape‐based membrane imprinting cannot. Glycosylation on enveloped viruses is structural and immunoevasive. The thick glycan shield of 22 N‐linked glycan sequons in SARS‐CoV‐2 spike protein monomers partially conceals immunodominant epitopes on the polypeptide backbone and promotes early attachment to host cell heparan sulfate proteoglycans and ACE2 (Angiotensin‐Converting Enzyme 2) [68, 81]. Host cell glycosylation machinery on viral protein sequences regulates viral glycan shield spatial density and monosaccharide composition, making them reproducible across virus strains and suited for systematic MIP imprinting [82]. Due to anionic phospholipid head groups and sialic acid‐bearing glycoproteins, enveloped viruses have a negative surface charge at physiological pH. However, variant‐specific alterations such as spike ectodomain mutations in SARS‐CoV‐2 Omicron subvariants can alter charge distribution and receptor binding [81, 83]. Drug delivery systems based on lipids purposefully have minimal surface protein landscapes. PEGylated liposomes and LNPs feature a protein‐repellent, glycan‐free shell that minimizes opsonization and extends circulation half‐life, and the PEG corona actively sterically restricts proteins from the lipid surface. Due to the PEG layer covering the ionizable lipid surface, well‐formulated mRNA‐LNPs exhibit a near‐neutral zeta potential at physiological pH, usually between −5 and −10 mV [84, 85]. Shape‐based recognition is needed for PEGylated LNP surfaces due to their geometric curvature and hydrophilic PEG corona. By contrast, EV and viral surfaces have a chemically rich, diverse exterior of proteins, glycans, and charged lipids that can interact in many ways.

#### Mechanical properties: Elasticity, curvature, and roughness

MIP recognition discussions often overlook vesicular surface mechanical properties such as membrane deformability under applied forces, bending rigidity, and surface roughness caused by protruding glycoproteins and compositional heterogeneity, which affect interface design [25]. All vesicular targets addressed here are soft, membrane‐bounded objects, not rigid nanoparticles, so their interaction with a polymer recognition cavity involves geometric fitting, chemical complementarity, and the membrane and polymer network's mechanical response to confinement and contact forces. Atomic force microscopy nanoindentation investigations have demonstrated that small extracellular vesicles contain Young's moduli of a few to several tens of megapascals, substantially lower than equivalent solid protein or polymer nanoparticles and strongly sensitive to membrane composition [86, 87]. Unsaturated phospholipids improve membrane fluidity and deformability, while cholesterol enrichment orders lipid packing and stiffness. The EV's bulk mechanical response and conformal contact with polymer recognition cavity walls depend on these species' balance [88]. Alterations in tetraspanin density in genetically engineered EV subpopulations caused nanomechanical response shifts in AFM (Atomic Force Microscopy) force spectroscopy experiments, suggesting that cell‐of‐origin‐specific protein‐lipid interactions within enriched microdomains contribute to the mechanical identity of the vesicle surface [86]. This mechanical heterogeneity among EV populations has been exploited diagnostically: Feng et al. reported that AFM‐based mechanical profiling of circulating EVs from patients with hematologic malignancies revealed nanomechanical signatures distinguishable from those of EVs from healthy donors, establishing that mechanical properties carry disease‐relevant information that complements surface protein and nucleic acid cargo analyses [89]. Membrane curvature, however related to elasticity, is equally important for MIP design. Cone‐shaped lipids like phosphatidylethanolamine, which enhance positive curvature by packing hydrophobic chains closer to head groups, are approximately two‐fold richer in small EV membranes compared to big ones. The target's outer surface is a sphere with a radius of curvature of 15–75 nm, so cavity walls must be tailored for productive, high‐avidity contact to collect small EVs. A rigid polymer matrix with cavity geometry defined against a flat template surface will have poor geometric complementarity to a highly curved vesicle. These observations indicate that vesicle deformability and curvature should be explicitly considered when designing the mechanical properties of the recognition interface. One last often ignored issue is surface roughness. Projecting glycoproteins, lipid domain boundaries, and membrane protein clusters give physiologically generated vesicles rough surfaces. Surface roughness affects how tightly a polymer recognition cavity can contact the vesicle surface, determining how well structural information from imprinting can be recovered during rebinding. The PEG brush gives engineered lipid nanoparticles a soft, fibrous texture, unlike protein‐decorated EV membranes, yet the absence of large transmembrane proteins and the homogenizing impact of the PEG corona decrease nanoscale topographic heterogeneity.

Taken together, the deformability, curvature, and surface heterogeneity of vesicular targets impose specific constraints on molecularly imprinted interfaces. Recognition sites should remain sufficiently accessible to accommodate intact vesicles while preserving the structural complementarity required for selective rebinding. Accordingly, surface‐accessible and thin recognition layers are particularly relevant for intact vesicle imprinting, as demonstrated in exosome‐imprinted sensing platforms [48, 49]. At the same time, excessive polymer rigidity may restrict conformal contact with soft vesicular surfaces, whereas excessive network flexibility may compromise cavity fidelity. Thus, vesicle‐imprinted interface design requires a balance between structural stability, target accessibility, and mechanical adaptability; however, these parameters have not yet been comprehensively optimized in intact vesicle MIP systems.

## MIP‐based sensors and analytical platforms for vesicular agent detection

### Optical biosensors: Surface Plasmon resonance coupled to MIP recognition layers

Surface plasmon resonance (SPR) is well suited to vesicular analyte detection because it enables label‐free and real‐time monitoring of binding events at metal–dielectric interfaces [90]. Resonant coupling of p‐polarized light to coherent oscillations of conduction electrons at a gold‐dielectric interface generates an evanescent electromagnetic field that decays exponentially into the neighboring medium with a penetration depth of 100–300 nm [90]. This decay length is ideal for small EVs: a 100‐nm vesicle collected at the sensor surface displaces its entire volume in the evanescent field and generates a mass‐per‐event contribution much greater than a protein biomarker of similar molar concentration. MIP recognition layers replace biological ligands on SPR chips, combining the imprinted cavity's programmable selectivity with this inherent physical sensitivity to create platforms capable of selective EV recognition while offering chemical stability and regenerability compared with protein‐based recognition elements.

The recognition performance of a MIP layer produced directly on an SPR gold chip depends on several phases. Template immobilization—cholesterol‐mediated insertion into a supported lipid bilayer, electrostatic adsorption, or oriented covalent coupling through surface‐expressed proteins—determines the template vesicle's sensor surface orientation, packing density, and imprinted cavity geometric fidelity [49]. When using whole EVs or intact viral particles instead of protein epitope surrogates, polymerization must preserve template structure. Before any biological application, template removal by sodium dodecyl sulfate washing, chaotropic solvent exposure, or enzymatic digestion must be confirmed by complete imprinting signal disappearance and MIP and NIP (Non‐imprinted polymer) responses across a selectivity panel [49, 91].

For intact‐EV imprinting on SPR chips, template quality is a critical determinant of recognition performance. In line with MISEV2023 recommendations [5], template EVs should be characterized before imprinting by particle sizing (e.g., nanoparticle tracking analysis), morphological imaging (e.g., transmission electron microscopy), and detection of EV‐associated markers such as CD63, CD81, CD9, and TSG101 [5]. Colorectal cancer cell lines such as HCT116 and HT29 are widely used EV sources, and their small EVs have been profiled in detail [92]. However, EVs from static monocultures may not reproduce the surface composition of tumor‐derived vesicles *in vivo*. Organ‐on‐a‐chip models offer a route to more physiologically relevant templates. The use of EVs collected from such models as imprinting templates for MIP–SPR sensors has not yet been reported and represents a prospective strategy for generating recognition cavities that better reflect the tumor microenvironment.

NanoMIP‐functionalized SPR platforms have also been developed for soluble cancer biomarkers and protein epitopes. Gür et al. used molecularly imprinted nanoparticles as recognition elements in an SPR biosensor for CA‐125 detection [93], while Erol et al. developed an epitope‐imprinted nanoMIP‐SPR platform targeting HER2 [94]. Although these studies do not involve vesicular targets, they are included here as background examples demonstrating the compatibility of nanoMIP recognition elements with SPR transduction. In contrast, whole‐particle recognition has been demonstrated using solid‐phase imprinted nanoMIPs immobilized on a portable SPR platform for the detection of intact SARS‐CoV‐2 particles (Fig. 3A) [95].

**Fig. 3:**
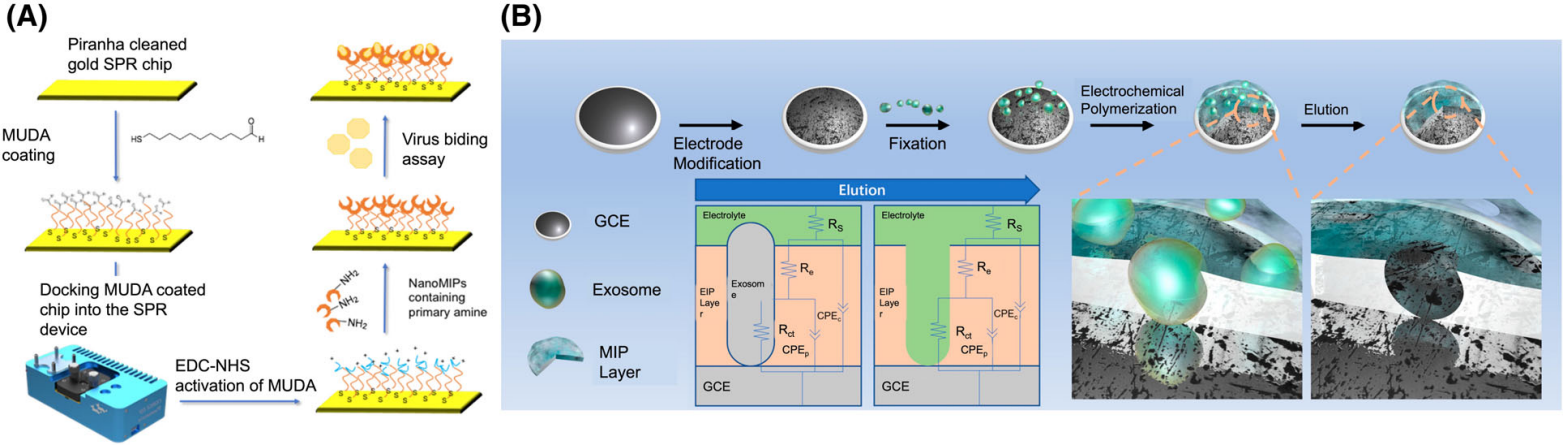
Representative MIP‐based sensing platforms for intact vesicular and enveloped viral particles. (A) Solid‐phase imprinted nanoMIPs immobilized on a portable SPR platform for the detection of intact SARS‐CoV‐2 particles. Reprinted with permission from [95]. (B) Schematic representation of the EIS (Electrochemical Impedance Spectroscopy) biosensor fabrication and the equivalent circuit model corresponding to the sensing process. Reprinted with permission from [49].

### Electrochemical MIP sensors for EVs and viruses

Electrochemical transduction complements optical sensing with lower instrumentation cost, direct compatibility with chip‐scale miniaturization, and electrical readout without optical alignment, making it attractive for point‐of‐care applications without laboratory infrastructure [96]. Impedimetric sensing is the most analytically productive electrochemical method for vesicular targets because it displaces several orders of magnitude more surface area per binding event than protein biomarkers of equivalent molar concentration, resulting in an intrinsically amplified impedance change without the need for excitation. Zhang et al. devised an impedimetric exosome sensor on a glassy carbon electrode for EV detection using cholesterol‐anchored template immobilization and *in situ* 3‐aminophenylboronic acid electropolymerization [49]. The boronic acid functional monomer covalently and reversibly bound to the exosome surface glycoproteins' cis‐diol groups, encoding the template's geometric shape and the imprinted cavity's glycan‐bearing surface proteins' spatial distribution. The sensor detected A549‐derived NSCLC (Non‐Small Cell Lung Cancer) exosomes with a detection limit of 2.03 × 10^3^ particles/mL, confirming selectivity against microvesicles, apoptotic bodies, and non‐malignant BEAS‐2B cells. The selectivity panel tested for cancer‐specific surface features rather than generic EV characteristics (Fig. 3B) [49]. Differences in glycoprotein arrangement and surface charge provide potential recognition cues for distinguishing A549‐derived exosomes from BEAS‐2B‐derived exosomes despite their overlapping sizes and shared tetraspanins.

Li et al. targeted bacterial OMVs using MIPs imprinted with lipopolysaccharide (LPS), a membrane component of Gram‐negative bacteria that is absent from mammalian EVs [97]. The thermodynamic basis for selective rebinding was the cyclic hydrogen bonding interactions between LPS's glucosamine and phosphate moieties and the MIP network's complementary functional groups, achieving OMV capture efficiencies from complex biological matrices that compared favorably with ultracentrifugation‐based enrichment while discriminating OMVs from different bacterial species based on LPS structural heterogeneity. This approach of using a lipid‐based surface component unique to the target vesicle class as the imprinting epitope rather than a protein marker that can change conformation during polymerization is broadly applicable and can be used to recognize other pathogen‐derived vesicle populations whose glycolipid composition differs from mammalian EVs.

MIP‐based electrochemical recognition has also been demonstrated for isolated viral surface proteins and epitopes, including SARS‐CoV‐2 spike glycoprotein and peptide fragments [80, 98, 99]. Although these studies do not involve intact vesicular or viral particles, they illustrate transferable strategies for recognizing membrane‐associated viral components.

### MIP‐based filtration and separation systems for vesicular agents

Device integration strategies using molecularly imprinted polymers in membrane and filtration formats differ from sensor platforms in operational goals. Sensors detect and quantify vesicular analytes at low concentrations in complex matrices, but MIP‐based membranes and filtration systems selectively enrich, separate, or deplete specific populations from larger sample volumes for bioanalytical sample preparation.

Molecularly imprinted composite membranes combine the selective binding of molecular imprinting with the mass transport of membrane filtration to capture targets during sample permeation [100]. In these systems, recognition sites can be incorporated throughout or at accessible regions of a porous membrane architecture rather than being restricted to a conventional planar sensing interface. Two general fabrication strategies have been widely described: the incorporation of pre‐synthesized MIP particles into porous support membranes, such as polyvinylidene fluoride, polyethersulfone, or cellulose acetate [100], and the direct formation of an imprinted polymer network within the membrane structure [101]. These studies establish the general architectural and fabrication principles of molecularly imprinted membranes but do not specifically address vesicular targets. Nevertheless, such membrane formats provide a potentially transferable framework for vesicle separation, provided that vesicle‐specific recognition elements and experimentally validated transport conditions are incorporated.

Direct evidence for vesicle separation with imprinted membrane formats is currently limited. Molecularly imprinted cryogel membranes have been applied to the isolation and adsorption of EVs, in which the interconnected macroporous structure allows intact vesicles to permeate while interacting with imprinted recognition sites (Section Imprinting strategies and challenges for vesicular agents) [50]. In addition, LPS‐imprinted polymers have shown that a membrane glycolipid unique to Gram‐negative bacteria can serve as an imprinting target for the selective recognition of OMVs (Section Electrochemical MIP sensors for EVs and viruses) [97]. Incorporating such recognition chemistry into membrane formats could, in principle, enable the enrichment of OMVs from matrices containing host‐derived EVs of overlapping size, which size‐based membranes cannot resolve; however, this has not yet been experimentally demonstrated. Systematic data on the capture efficiency, selectivity, and throughput of imprinted membranes for vesicular targets therefore remain an important need.

### Microfluidic integration of MIP‐based vesicular agent recognition

#### Rationale, mass transport considerations, and channel design

It should be noted at the outset that experimental studies integrating MIP recognition with microfluidic handling of intact vesicular targets remain scarce. Most of the microfluidic studies discussed in this section therefore do not involve molecular imprinting. They are included because they define the transport, pre‐concentration, and integration conditions under which a vesicle‐imprinted interface would have to operate, and they are explicitly identified as such. Direct MIP–microfluidic demonstrations are distinguished from these enabling technologies wherever available.

Sample supply and processing conditions limit the performance of even the most selective MIP recognition interface. Plasma, urine, nasopharyngeal wash, and cerebrospinal fluid are clinical specimens relevant to EV‐based diagnosis, virus detection, and drug quality monitoring, as the target vesicular population is present in a complex matrix at low or variable concentrations, and traditional isolation methods such as differential ultracentrifugation, size exclusion chromatography, and immunomagnetic precipitation are time‐consuming, equipment‐intensive, and require frequent repetition. In contrast to macroscale processing, microfluidic systems confine sample handling to precisely engineered channel geometries at the nano‐ to microliter scale, where characteristic length scales govern mass transport and separation physics [102, 103].

Due to the nanoscale dimensions of EVs and viral particles, their diffusion coefficients in aqueous solution are extremely low (10^−12^ m^2^/s for a 100 nm particle). This means that transporting a significant fraction of sample particles to a planar sensor surface from a few micrometers away takes longer than the diagnostic timescale [104]. Microfluidic channels alleviate this constraint by using convective delivery to convey analyte continuously to the recognition surface at a rate that scales with channel shape and flow velocity rather than particle diffusivity [102]. The geometric architecture of the microchannel around the MIP recognition surface, as well as the recognition layer chemistry, determines platform performance. Herringbone groove patterns machined into the channel ceiling create chaotic advective mixing that continuously folds streamlines across the channel cross‐section, dramatically increasing vesicle surface contact and capture efficiency from a few percent in smooth‐wall channels to 50–80% under optimized mixing conditions [105]. Using inertial focusing forces, spiral channel designs concentrate particles laterally before they reach the identification zone, bringing a pre‐concentrated EV population to the MIP surface under regulated hydrodynamic exposure. These geometries have not yet been combined with vesicle‐imprinted surfaces, but they directly address the diffusion limitation discussed in Section Binding kinetics of MIPs targeting vesicular agents.

#### Integrated microfluidic platforms for EV isolation and detection

The sequential treatment of isolation and detection on separate equipment with manual sample transfer between phases is the main constraint of current EV diagnostic procedures. This architecture causes analyte loss at each transfer, contamination risk, and inter‐laboratory heterogeneity that hinders clinical standardization. As engineering tools have become more accessible, integrated microfluidic platforms that isolate and detect EVs in a single workflow have advanced significantly [106]. Although neither of the following studies used MIP recognition, they illustrate the integration steps required for vesicle‐imprinted platforms. Wang et al. developed a filter‐electrochemical microfluidic chip that eliminated the need for off‐chip ultracentrifugation by combining size‐based nanoporous membrane filtration with *in situ* electrochemical quantification of EV surface proteins from whole blood [107]. Talebian Gevari et al. integrated electrokinetic pre‐concentration with downstream electrochemical detection to show that dielectrophoretic force concentrated EVs at a defined location in the channel before routing them to a recognition surface, increasing the detection limit by over an order of magnitude compared to passive flow‐through delivery [108]. The field is moving toward end‐to‐end integration, from biologically relevant EV source to recognition interface to analytical readout.

The earliest direct demonstration of whole‐particle MIP recognition within a microfluidic device was reported by Birnbaumer et al., who integrated virus‐imprinted polymer layers with contactless capacitive microsensors in a microfluidic biochip. Tobacco mosaic virus was selectively detected against human rhinovirus, and bound particles could be released by increasing the flow velocity, enabling sensor regeneration and reuse [109]. Because both viruses are non‐enveloped, this study is included only as a comparative example of flow‐integrated whole‐particle imprinting rather than as direct vesicle recognition. Nevertheless, it demonstrates how microfluidic flow can be used to control particle delivery and particle–MIP interactions within an integrated recognition platform. Direct demonstrations combining microfluidic handling with molecularly imprinted interfaces for intact vesicular targets remain scarce.

### Lipid‐based drug delivery systems: Current status and prospects for MIP–microfluidic integration

Microfluidic technologies are already well established in the field of lipid‐based drug delivery, primarily for the controlled and reproducible synthesis of liposomes and LNPs and for the evaluation of their performance [110]. In parallel, microfluidic resistive pulse sensing (MRPS) enables single‐particle size and concentration measurements of nanoscale particles in complex fluids and has been standardized for measuring EVs together with other particles in biofluids [111]. These platforms, however, characterize lipid‐based vesicles by physical parameters such as size and concentration and do not provide chemical selectivity toward specific surface compositions.

To our knowledge, MIP‐based recognition of intact liposomes or LNPs within microfluidic devices has not yet been experimentally reported, which represents a clear gap relative to the EV and virus applications discussed above. The closest experimental evidence comes from phospholipid‐oriented imprinting, which has shown that membrane lipid head groups can be imprinted and used for the selective recognition of plasma membranes and exosomes [22, 47]. Because liposomes and LNPs are composed of defined phospholipid, cholesterol, and PEG‐lipid components, membrane‐component imprinting represents a potentially transferable route toward the selective recognition of lipid‐based carriers, although the PEG corona of clinical LNP formulations may restrict access to lipid head groups (Section Lipid bilayer composition). Combining such imprinted interfaces with established microfluidic synthesis or characterization workflows could, in principle, add compositional selectivity to physical particle analysis. However, this remains a prospective direction that requires experimental validation, including demonstration of selectivity against formulation variants and of interface stability in lipid‐rich media. Representative MIP‐based systems reported for the recognition of intact vesicular agents or vesicle‐specific membrane components are summarized in Table 1.

**Table 1:** Representative MIP‐based systems that recognize intact vesicular agents or vesicle‐specific membrane components.

Target system	Template/target	MIP strategy	Platform	Key performance	Main feature/limitation	References
Cells and exosomes	Membrane phospholipids	Phospholipid (membrane‐component) imprinting	Selective recognition/capture	Selective recognition of cell and exosome surface	Recognition through a membrane lipid component rather than whole‐vesicle imprinting	[22]
Exosomes	Intact exosomes	Surface imprinting with post‐imprinting modifications	Fluorescence sensing on MIP‐coated substrate	Pretreatment‐free sensing of intact exosomes	Direct intact‐exosome imprinting	[48]
A549‐derived exosomes	Intact exosomes	Whole‐vesicle surface imprinting; APBA‐based electropolymerization	Electrochemical impedance sensing (EIS)	LOD (Limit of Detection): 2.03 × 10^3^ particles·mL^−1^ selective against microvesicles, apoptotic bodies, and BEAS‐2B‐derived exosomes	Direct intact‐EV recognition; discrimination from other vesicular populations	[49]
Extracellular vesicles	Intact EVs	Molecularly imprinted cryogel membrane	Cryogel membrane‐based isolation and adsorption	Adsorption capacity 1075 particles·g^−1^ cryogel at pH 5.0; Langmuir‐type adsorption; regenerable with 1 M NaCl	Macroporous architecture allowing permeation and capture of intact vesicles	[50]
Bacterial outer membrane vesicles (OMVs)	Lipopolysaccharide (LPS) on OMV surface	LPS‐imprinted MIP	Selective recognition/capture	Capture from complex biological matrices; discrimination between OMVs from different bacterial species	Vesicle recognition through a membrane‐specific glycolipid epitope rather than whole‐vesicle imprinting	[97]
SARS‐CoV‐2	Inactivated intact SARS‐CoV‐2 particles	Solid‐phase imprinted nanoMIPs	Portable SPR	Detection at 10^4^–10^6^ PFU·mL^−1^; selectivity over influenza A	Direct whole‐particle recognition using nanoMIP‐SPR	[95]
SARS‐CoV‐2	Intact viral particles	Whole‐virus imprinting	Electrochemical sensing	LOD: 0.1 fM	Direct whole‐particle recognition with selectivity against H5N1	[112]

## Conclusion and future perspectives

Selective recognition of nanoscale vesicular agents, including extracellular vesicles, membrane‐enveloped viruses, and lipid‐based drug delivery systems presents one of the defining analytical challenges at the intersection of biomedical diagnostics, pharmaceutical quality control, and infectious disease surveillance. This review has examined how molecularly imprinted polymers have evolved from tools for small‐molecule recognition into sophisticated platforms capable of engaging the complex, dynamic, and heterogeneous surfaces that characterize membrane‐bounded nanostructures. The field has advanced considerably. Surface‐accessible imprinting, membrane‐component imprinting, and thin or macroporous recognition architectures have made it possible to generate imprinted interfaces for intact or membrane‐derived vesicular targets that are difficult to address with conventional bulk imprinting. Integration with optical and electrochemical transducers has enabled label‐free detection of exosomes and intact viral particles, and individual studies have demonstrated selectivity against other vesicle populations or related viruses [95, 112]. However, MIP‐based recognition of intact liposomes or LNPs has not yet been experimentally demonstrated and remains a prospective direction, as discussed in Section Lipid‐based drug delivery systems: Current status and prospects for MIP–microfluidic integration. Accordingly, significant challenges must be overcome before MIP‐based vesicular recognition can transition from proof‐of‐concept demonstrations to routine clinical and industrial deployment.

The most pressing challenge confronting the field is the necessity of standardization. In contrast to small‐molecule targets, which are characterized by their well‐defined molecular structures, vesicular agents exhibit a greater variability in terms of size, composition, and surface architecture. This variability is influenced by the biological source, the method of isolation, and the storage conditions. The MISEV2023 guidelines [5] have established minimum standards for EV characterization; however, no comparable framework exists for evaluating the recognition of synthetic receptors by vesicular targets. To facilitate the comparison of MIP selectivity panels across different laboratories, the field must adopt standardized reference materials, well‐characterized EV populations, viral pseudoparticles, and lipid nanoparticle formulations with clearly defined specifications. Without such standards, meaningful comparison of reported detection limits and selectivity factors remains difficult, which impedes both scientific progress and regulatory translation.

The reproducibility of MIP synthesis itself presents related difficulties. It is evident that the variability observed in binding‐site density, cavity geometry, and non‐specific binding capacity across different batches remains above acceptable thresholds for clinical translation. Controlled radical polymerization techniques, automated solid‐phase synthesis protocols, and computational pre‐screening of monomer–template interactions [31, 113] have begun to address this limitation. However, broader adoption of quality‐controlled synthesis workflows will be essential as the field moves toward commercial applications.

Although recent advances have narrowed the selectivity gap between MIPs and biological recognition elements, achieving antibody‐level discrimination among closely related vesicular populations remains challenging. The structural overlap between tumor‐derived and non‐malignant EVs, between pathogenic viruses and host‐derived vesicles, and between correctly assembled LNPs and formulation variants demands recognition interfaces capable of integrating multiple orthogonal binding criteria: geometric fit, charge complementarity, glycan recognition, and mechanical response within a single imprinted cavity. The emerging concept of “plastic antibodies,” in which nanoMIP particles functionalized with multiple epitope‐complementary regions mimic the multivalent binding geometry of immunoglobulins, represents a promising direction [58, 61]. Further development of hierarchically organized recognition interfaces that combine lipid‐targeting, protein‐targeting, and glycan‐targeting functional monomers within spatially defined domains may ultimately enable the discrimination of vesicular subpopulations that remain inaccessible to current single‐epitope approaches.

A conceptual frontier concerns the dynamic nature of vesicular surfaces and the corresponding need for recognition interfaces capable of adaptive response. Biological membranes are not static templates; they undergo continuous lipid diffusion, protein clustering, and compositional remodeling. The mechanical compliance of vesicle membranes, reflected in Young's moduli that are substantially lower than those of rigid nanoparticles [114, 115], suggests that binding events can induce conformational changes in both the target and the recognition cavity. MIP designs that exploit this softness through controlled network flexibility, rather than treating it as a source of noise, may achieve recognition modes inaccessible to rigid synthetic receptors. The balance between structural memory and adaptive response represents a design parameter that has been empirically explored, but not yet systematically optimized.

For diagnostic applications, the pathway to clinical translation requires validation at scales and in patient populations that remain largely unexplored. Diagnostic MIP‐based EV platforms reported to date have been evaluated largely with cell line‐derived material and controlled matrices. Prospective clinical studies are needed to compare MIP‐based EV diagnostics with established biomarkers in relevant disease cohorts, thereby establishing their clinical utility. The inherent stability of MIPs—including thermal resistance, solvent compatibility, and regenerability—makes them promising candidates for point‐of‐care deployment in resource‐limited settings, where cold‐chain infrastructure and trained personnel may be lacking. However, this potential has so far been demonstrated mostly in principle rather than in practice.

For pharmaceutical applications, molecular imprinting of intact liposomes and LNPs remains a prospective rather than experimentally established application. The increasing need for in‐process characterization of lipid‐based nanocarriers provides a potential opportunity for future MIP interfaces capable of distinguishing formulation‐dependent surface features. Integration of such recognition interfaces with continuous manufacturing workflows may eventually complement existing analytical methods, but direct experimental validation of intact‐LNP imprinting is still required.

Integrating MIP recognition interfaces with microfluidic processing opens powerful new possibilities for analyzing vesicular agents. By guiding samples through carefully designed microchannels, these platforms can overcome the mass transport limitations that often reduce detection sensitivity in conventional, larger‐scale systems. In non‐imprinted EV devices, microfluidic features such as herringbone mixers and electrokinetic pre‐concentration have been shown to increase vesicle–surface contact [103, 108]. Bringing these elements together into fully integrated platforms which are capable of isolation, recognition, and quantification within a single automated workflow could help minimize sample loss, reduce contamination risk, and improve consistency between laboratories, all of which are key challenges for clinical standardization.

Several emerging directions warrant particular attention. First, the convergence of MIP technology with machine learning approaches for both synthesis optimization and signal interpretation may accelerate the design cycle and improve performance prediction. Second, multiplexed recognition interfaces capable of simultaneously detecting and discriminating multiple vesicular populations within a single sample would address the diagnostic complexity of conditions such as cancer, where tumor‐derived EVs coexist with EVs from stromal, immune, and endothelial cells. Third, the extension of MIP recognition to prokaryotic vesicle populations, outer membrane vesicles from pathogenic bacteria, for instance, opens applications in infectious disease diagnostics and microbiome research that have received limited attention relative to eukaryotic EVs and mammalian viruses.

MIPs have matured from a molecular recognition curiosity into a versatile platform technology with demonstrated applicability to some of the most challenging targets in bioanalytical science. The vesicular agents considered here—including extracellular vesicles, membrane‐enveloped or membrane‐associated viral particles, and lipid‐based nanocarriers—share membrane‐associated structural features that require recognition strategies distinct from those developed for small molecules or rigid macromolecules. The field has responded with innovations in imprinting chemistry, polymer architecture, and device integration that position MIPs as promising candidates to complement biological recognition elements for these targets. Realizing this potential will require sustained attention to standardization, clinical validation, and manufacturing reproducibility challenges that are organizational and translational as much as scientific. The foundations reviewed here suggest that the selective recognition of vesicular agents by rationally engineered polymeric interfaces is a realistic but not yet realized objective whose realization will depend on the coordinated efforts of polymer chemists, bioanalytical scientists, clinicians, and regulatory bodies in the years ahead.

## Conflict of interest

The authors declare no conflict of interest.

## Author contributions

All authors (ÖE, EGY, SG, NK and FI) contributed to the writing of the manuscript. FI supervised the work.
